# Enhanced tumorigenicity by mitochondrial DNA mild mutations

**DOI:** 10.18632/oncotarget.3698

**Published:** 2015-03-30

**Authors:** Alberto Cruz-Bermúdez, Carmen G. Vallejo, Ramiro J. Vicente-Blanco, María Esther Gallardo, Miguel Ángel Fernández-Moreno, Miguel Quintanilla, Rafael Garesse

**Affiliations:** ^1^ Instituto de Investigaciones Biomédicas “Alberto Sols”, Consejo Superior de Investigaciones Científicas (CSIC), Universidad Autónoma de Madrid (UAM), Madrid, Spain; ^2^ Departamento de Bioquímica and Centro de Investigación Biomédica en Red en Enfermedades Raras (CIBERER), Facultad de Medicina, UAM, Madrid, Spain; ^3^ Instituto de Investigación Sanitaria Hospital 12 de Octubre (i+12), Madrid, Spain; ^4^ Instituto de Investigación Hospital Universitario La Paz (IdiPAZ), Madrid, Spain

**Keywords:** cancer, mitochondria, mtDNA mutations, LHON, retrograde signaling

## Abstract

To understand how mitochondria are involved in malignant transformation we have generated a collection of transmitochondrial cybrid cell lines on the same nuclear background (143B) but with mutant mitochondrial DNA (mtDNA) variants with different degrees of pathogenicity. These include the severe mutation in the tRNA^Lys^ gene, m.8363G>A, and the three milder yet prevalent Leber's hereditary optic neuropathy (LHON) mutations in the *MT-ND1* (m.3460G>A), *MT-ND4* (m.11778G>A) and *MT-ND6* (m.14484T>C) mitochondrial genes. We found that 143B ρ^0^ cells devoid of mtDNA and cybrids harboring wild type mtDNA or that causing severe mitochondrial dysfunction do not produce tumors when injected in nude mice. By contrast cybrids containing mild mutant mtDNAs exhibit different tumorigenic capacities, depending on OXPHOS dysfunction.

The differences in tumorigenicity correlate with an enhanced resistance to apoptosis and high levels of NOX expression. However, the final capacity of the different cybrid cell lines to generate tumors is most likely a consequence of a complex array of pro-oncogenic and anti-oncogenic factors associated with mitochondrial dysfunction.

Our results demonstrate the essential role of mtDNA in tumorigenesis and explain the numerous and varied mtDNA mutations found in human tumors, most of which give rise to mild mitochondrial dysfunction.

## INTRODUCTION

Cancer is a complex disease triggered by a variety of factors, including mutations in proto-oncogenes and tumor suppressor genes. As a consequence, cancer cells deregulate their cell cycle and impair apoptosis, promoting a higher proliferation rate that leads to the accumulation of a cell mass [1]. A key feature of cancer cells is their ability to reprogramme metabolism in order to adapt it to rapid proliferation and exponential growth. This is achieved by increasing aerobic glycolysis, the well-known Warburg effect, which supplies the metabolic intermediates used in anabolic processes [2, 3]. In addition, oxidative phosphorylation (OXPHOS) is downregulated in most cancer cells, the combination of high glycolysis and low OXPHOS (the bioenergetic signature) becoming a feature used to diagnose early stages of cancer and as a marker of tumor progression [4].

The discovery of altered mitochondria in tumor tissues has stimulated studies on the relationship between mitochondria and cancer [5-7]. Accordingly, mutations in nuclear genes encoding enzymes of the Krebs cycle have been found in hereditary paragangliomas, pheochromocytomas and among other human tumors [8-10]. In addition, the recent massive sequencing of tumor and non-tumor tissues pairs performed by The Cancer Genome Atlas (TCGA) consortium identified numerous mutations in the mtDNA of cancer cells, although vast majority seems to provoke non severe dysfunctions. This suggests that mtDNA variants could drive metabolic alterations that are important in the malignant transformation, although the full pathological significance of these observations remains unclear [11-13].

In normal cells, most cellular ATP is produced in the mitochondria by the OXPHOS system, which is comprised of the electron transport chain complexes (plus two electron carriers: coenzyme Q and cytochrome c) and the multimeric ATP-synthase (complex V). OXPHOS biogenesis requires the coordinated expression of two genomes, the nuclear and mitochondrial. Mitochondrial DNA (mtDNA) encodes a limited but essential number of genes for OXPHOS biogenesis, including the RNA components of the translational apparatus, two rRNAs (12S and 16S) and twenty two tRNAs, along with thirteen proteins, all of which are subunits of the I, III, IV and V complexes of the OXPHOS system. Due to their dual genetic control, OXPHOS alterations (which provoke the so-called OXPHOS diseases or mitochondrial diseases) can be caused by mutations in mitochondrial and/or nuclear DNA. The mtDNA accumulates mutations, many of them cause moderate or no phenotypic effects, while others cause severe diseases [14].

Human mitochondrial diseases are characterized by a wide variety of symptoms and they affect different organs. To date, over 250 pathogenic mutations have been identified within the human mitochondrial genome affecting genes encoding rRNAs, tRNAs and proteins. mtDNA mutations may cause multisystemic syndromes, such as in most tRNA mutations, or they may affect specific organs [14]. A paradigmatic tissue-specific mitochondrial disease is Leber's hereditary optic neuropathy (LHON), caused by non-severe mutations in genes encoding complex I subunits that provoke severe visual impairment due to retinal ganglion cell death. Mutations in three polypeptides are responsible for 90-95% of all cases of LHON: *MT-ND1* (m.3460G>A), *MT-ND4* (m.11778G>A) and *MT-ND6* (m.14484T>C) [15, 16].

In addition to their role in cellular energy production, mitochondria are metabolic signaling centers that fulfil a variety of essential functions including apoptosis, ROS production and calcium homeostasis in different cells and tissues [17]. However, the fundamental molecular mechanisms underlying these processes, which are critical to understand the role of mitochondria in health and disease, are mostly unknown. Cytoplasmic hybrids, also known as transmitochondrial cybrids or cybrids, represent models that are being used widely to study the effects of mtDNA variants on cell physiology and human pathology [18, 19]. These cells are generated by fusing mtDNA depleted cells (ρ^0^ cells) with cytoplasts, typically platelets or enucleated fibroblasts [20].

The first studies into cancer involving mtDNA variants were done before the development of ρ^0^ cells and therefore, they were prior to the currently available cybrid technology. In those studies, the tumorigenic properties of a cell line were modified by altering its cytoplasmic content [21, 22]. Subsequently, cybrid technology has been used to examine the relationship between mtDNA and tumorigenicity in different cell lines and for a variety of mtDNA mutations. These studies suggested different mechanisms are at play in tumor development, involving changes in ROS levels, Hif-1α stabilization, sensitivity to apoptosis, etc., but not in a conclusive manner [23-29]. In addition, the literature is not always coherent regarding such correlations, in most cases because these analyses studied the effect of a unique mutation in reference to a unique control and in only one cybrid clone.

To clarify this controversy, in this work we have analyzed a group of parameters in at least two clones of every cell line of a wide panel of 143B osteosarcoma-derived cybrids harboring several mtDNA mutations and their corresponding controls.

Our results clearly demonstrate that mtDNA genetic variants modulate the tumorigenicity of K-RAS transformed 143B osteosarcoma cells. The mtDNAs that render a functional OXPHOS and mutant mtDNAs that severely disrupt OXPHOS all suppress tumorigenicity, as does the depletion of mitochondria in 143B ρ^0^ cells. However, mtDNA mutations that impair OXPHOS but do not produce a loss-of-function all support tumorigenesis. Furthermore, in the homogeneous system of cybrid cell lines, the tumorigenic potential is directly correlated with the degree of OXPHOS impairment. This would explain the high number and variety of mtDNA mutations accumulation found in human tumors. Differences in the tumorigenic potential of 143B cybrids are correlated with resistance to apoptosis and strong NOX expression, which is most likely modulated by a complex array of pro-oncogenic and anti-oncogenic factors derived from mitochondrial dysfunction.

## RESULTS AND DISCUSSION

### The 143B cell line requires mtDNA to induce tumor formation

**Figure 1:**
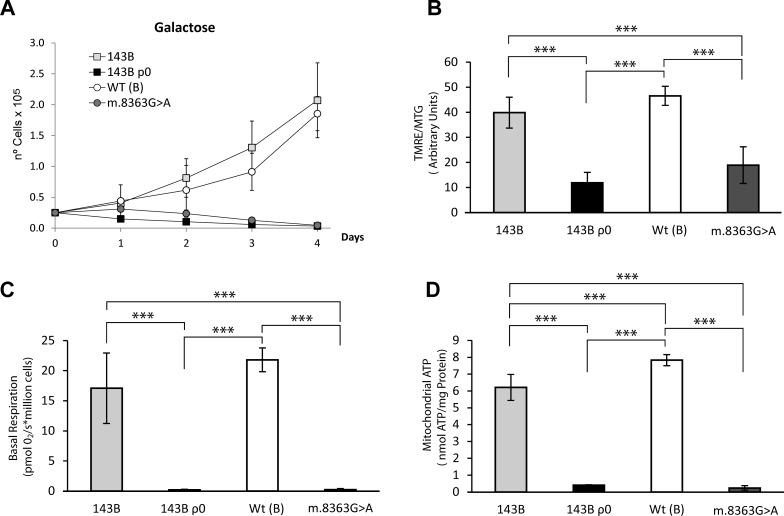
OXPHOS functional characterization of the different cell lines (**A**) Growth curves in 0.9 g/L galactose showing severe OXPHOS dysfunction in the 143B ρ^0^ and m.8363G>A cell line. (**B**) Mitochondrial inner membrane polarization was evaluated as the ratio of Tetramethyl rhodamine ester (TMRE) to MitoTracker Green (MTG) assessed by flow cytometry. (**C**) Basal respiration. Oxygen Consumption was measured using the Oroboros oxygragh-2k. (**D**) Steady state mitochondrial ATP. Adenosine Triphosphate (ATP) was detected using the luminometric luciferin-luciferase method in cybrid samples incubated for 2h with 5mM 2-deoxy-D-glucose/1mM pyruvate. At least three independent experiments were measured for each parameter and the data represent the means and standard deviations. The haplogorup B of wild type is in parenthesis. The mean from two independent m.8363G>A clones is also shown. The data was analyzed using one way ANOVA and Tukey's multiple comparison test, and a P-value <0.05 was considered significant: *=P ≤ 0.05, **=P ≤ 0.01, ***=P ≤ 0.001.

The 143B cell line, generated by transforming TE85 human osteosarcoma cells with a K-ras oncogene, has been used in numerous studies as a cancer model given its ability to efficiently form tumors in nude mice, exhibiting considerable cell motility and invasive potential [30, 31]. More than twenty years ago a 143B TK^−^ cell line was successfully used to generate a line devoid of mtDNA, the so called 143B ρ^0^ cell line, which was also functionally repopulated with mitochondria from donors [20]. The parental 143B cells contain a mtDNA molecule that belongs to haplogroup X and that harbors the homoplasmic m.6267G>A mutation in the cytochrome oxidase I (CO1) subunit, a mutation that impairs cytochrome c oxidase (COX) activity and respiration [32]. The m.6267G>A mutation has been associated with different types of cancer but it has yet to be associated with mitochondrial diseases. This is probably due to its weak impact on the OXPHOS function as revealed by its slightly decrease in MIMP (Mitochondrial Inner membrane Potential), ATP levels and oxygen consumption that do not affect their ability to grow in galactose (Figure 1). As expected, there was no mitochondrial activity in these 143B ρ^0^ cells.

**Figure 2:**
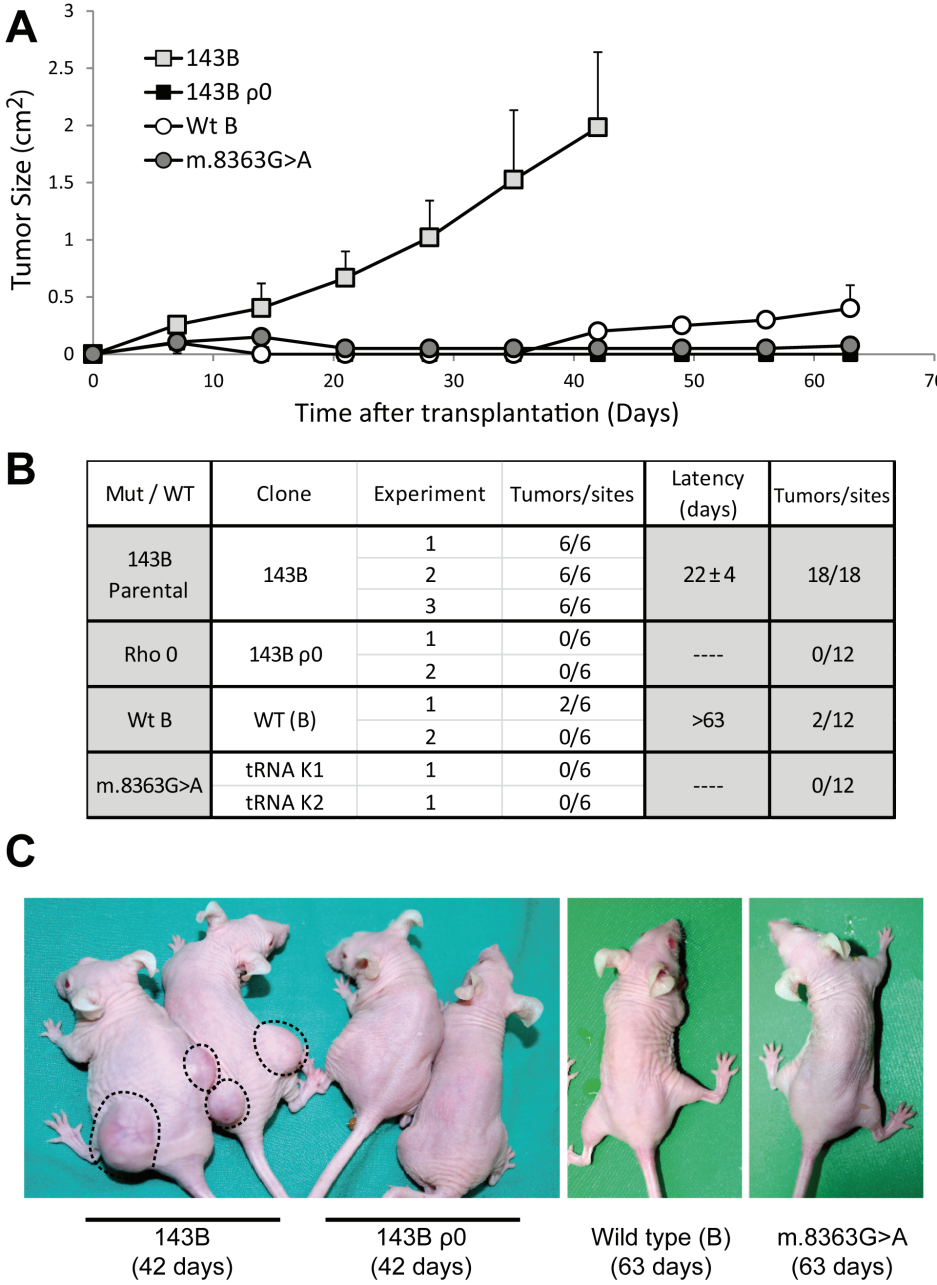
The 143B osteosarcoma cell line exerts its tumorigenic potential through the mild mutation m.6267G>A (**A**) Tumor growth induced by injection of 5×10^6^ cells at each site, expressed as the mean for all the tumors generated by each genotype ± standard deviation. (**B**) Summary of the different clones and experiments carried out. The table records the number of tumors developed larger than 0.25 cm^2^ at the end of the experiment relative to the total number of injection sites. The latency period was defined as the time needed for the tumors to reach a size of 0.75 cm^2^. (**C**) Representative images of mice injected with the different cell lines. Note the difference in tumor growth in animals injected with 143B cells. The days after the injection are shown in parentheses.

The importance of mtDNA in the capacity of 143B cells to produce tumors was investigated by injecting 5 × 10^6^ 143B or 143B ρ^0^ cells into nude mice. The 143B cells produced tumors at all injection sites within a relatively short period of 22 days (Figure 2), tumors that exhibited a high growth rate and that reached a size of 2 cm^2^ in 42 days. By contrast, 143B ρ^0^ cells were unable to generate tumors, in accordance with the non-tumorigenic nature of other ρ^0^ cell lines [33] but in contrast to the proposed enhanced tumorigenicity of 143B ρ^0^ cells due to their anchorage independent growth [34]. These results demonstrate that the capacity of 143B cells to produce tumors is strictly dependent on the presence of mtDNA.

### mtDNA rendering a functional or non-functional OXPHOS system do not support tumorigenicity

To explore the possibility that mtDNA variants may modulate the tumorigenic properties of transformed cells, we investigated the tumorigenicity of cybrids harboring the well-known, severe mutation m.8363G>A. The m.8363G>A mutation in the mitochondrial tRNA^Lys^ gene severely impairs the synthesis of mitochondrial proteins, yielding a non-functional respiratory chain [18]. Like 143B ρ^0^ cells, cybrids repopulated with mtDNA harboring the m.8363G>A mutation do not respire or produce mitochondrial ATP, and they therefore die in galactose medium (Figure 1). However, these cells are not tumorigenic (Figure 2), indicating that not only the absence of mtDNA but also a severe mtDNA mutation that fully impairs OXPHOS function acts as a tumor suppressor. Interestingly, control cybrids containing wild type mtDNA also showed weak tumorigenic behavior, inducing only small tumors at less than 20% of the injection sites, with slow growth rates and long latencies (above 63 days, Figure 2). These results suggest that the specific mtDNA in 143B cells is essential to define their tumorigenicity. Thus, mtDNAs that render either fully functional or non-functional OXPHOS systems do not support tumorigenicity, in contrast to the m.6267G>A mutation that only mildly impairs OXPHOS function.

### mtDNA harboring mild mutations supports tumorigenicity in function of the degree of OXPHOS impairment

To investigate the relationship between mild mtDNA mutations and tumorigenicity, we generated cybrids harboring the prevalent LHON mutations and their respective control cell lines. Studies into the pathological consequences of LHON mutations show that the same homoplasmic mutation may affect some individuals (even leading to blindness) while not affecting others at all [16]. Therefore, prevalent LHON mutations are excellent examples of mild mtDNA alterations that affect OXPHOS but that do not necessarily give rise to significant phenotypic manifestations, such as the m.6267G>A.

**Figure 3:**
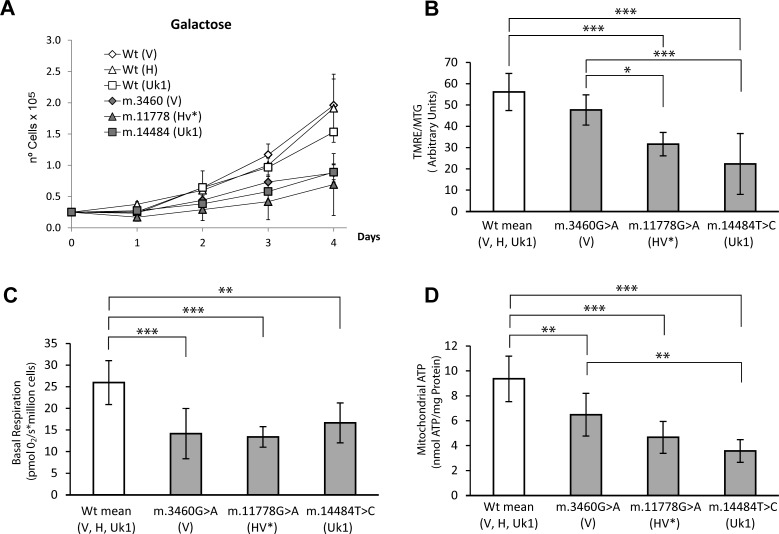
Functional characterization of the different LHON cybrid cell lines and their controls (**A**) Growth curves in 0.9 g/L galactose (**B**) Mitochondrial inner membrane polarization was evaluated as the Tetramethyl rhodamine ester (TMRE) to MitoTracker Green (MTG) ratio, assessed by flow cytometry. (**C**) Basal respiration. Oxygen Consumption was measured using the Oroboros oxygragh-2k. (**D**) Steady state Mitochondrial ATP in cybrid cells. ATP was detected using the luminometric luciferin-luciferase method in cybrid samples incubated for 2h with 5mM 2-deoxy-D-glucose/1mM pyruvate. The data for cybrids harboring mtDNA mutations are the mean of two clones and the three cybrid clones harboring wild type molecules were grouped. Each clone was measured in at least three independent experiments for each parameter and the mtDNA haplogroups are showed in parentheses. The data are the means ± standard deviation, and they were analyzed using one way ANOVA and Tukey's multiple comparison test, with a P-value <0.05 considered to be significant: *=P ≤ 0.05, **=P ≤ 0.01, ***=P ≤ 0.001.

Thus, we repopulated 143B ρ^0^ cells with mtDNA harboring the well characterized mutations m.3460G>A (*MT-ND1*), m.11778G>A (*MT-ND4*) and m.14484T>C (*MT-ND6*), which belong to V, HV* and Uk1 haplogroups, respectively as well as wild type mtDNAs for each of those haplogroups. Since the biochemical parameters and *in vitro* growth of cells harboring the different wild type versions of mtDNA (V, H and Uk1) were comparable, they were grouped in a single category (Figure 3). Compared to the wild type cells, cybrids harboring mtDNAs carrying LHON mutations showed reduced MIMP, basal respiration and mitochondrial ATP, as well as impaired growth in galactose. Thus, the functional impact of the three LHON mutations on the OXPHOS system was moderate, as reported previously [16].

**Figure 4:**
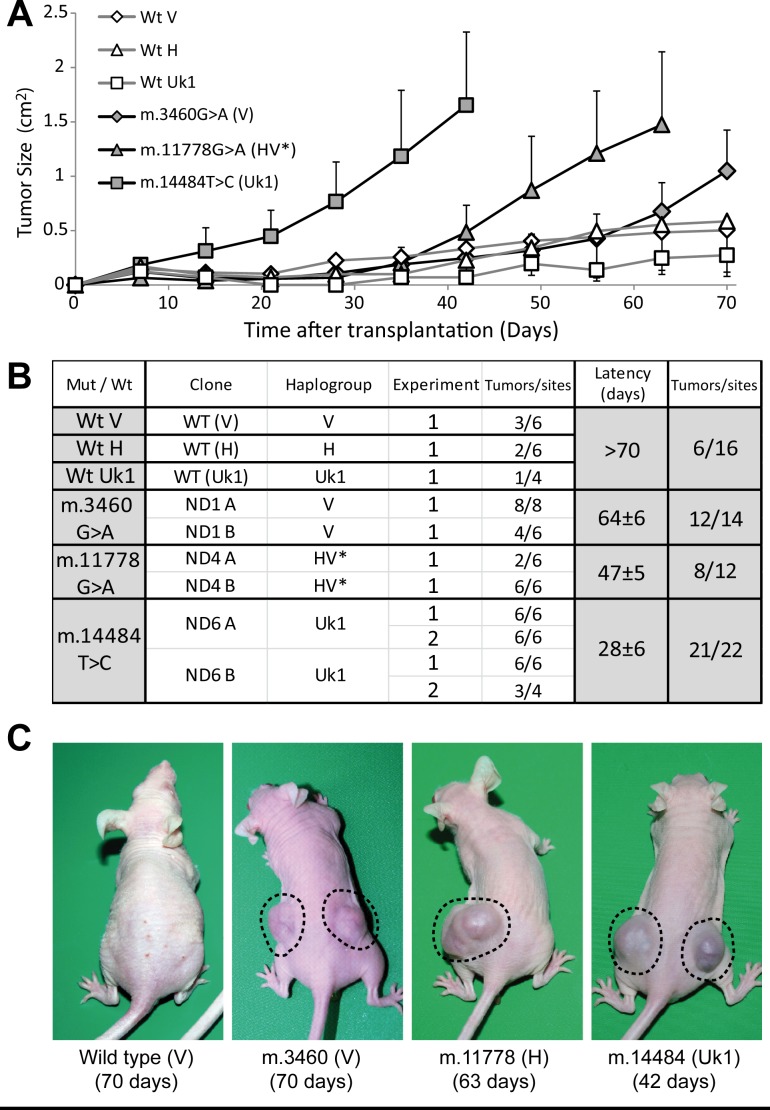
Mild mtDNA mutations are required for tumorigenicity (**A**) Tumor growth induced upon injection of 5×10^6^ cells at each site, expressed as the mean for all the tumors generated of each genotype ± standard deviation. (**B**) Summary table with the different clones and experiments carried out. The table records the number of tumors developed (larger than 0.25 cm^2^) relative to the total number of injection sites at the end of the experiment. The latency period was defined as the time needed for tumors to reach a size of 0.75 cm^2^. (**C**) Representative images of mice injected with the different cell lines, with the days after the injection indicated in parentheses.

Injection of nude mice with the cybrid lines demonstrated that the different mtDNA genetic variants confer variable tumorigenicity to the 143B nuclear background (Figure 4). All LHON cybrids were tumorigenic, although to a different extent. Cybrids containing the m.14484T>C mutation rapidly produced tumors at 95% of injection sites within 28 days, a behavior similar to that of the parental 143B cells (Figure 2). The tumorigenic potential of m.11778G>A cybrids was more moderate, inducing tumors at 67% of injection sites after about 47 days, although once formed these tumors grew very fast. Finally, there was a high incidence of tumors in cybrids harboring the m.3460G>A mutation (86% of sites) but with a long latency of 65 days and a slow growth rate. In contrast to these mutants, wild type V, H, Uk1 cybrids were weakly tumorigenic inducing small tumors at less than 40% of injection sites with a very slow growth rate and a latency above 70 days, consistent with the behavior of cybrids containing wild type mtDNA of the haplogroup B (Figure 2).

Remarkably, the tumorigenic behavior of LHON cybrids was correlated with the degree of OXPHOS dysfunction exhibited by the cell lines. Thus, the m.3460G>A mutation was associated with the lowest tumorigenicity and OXPHOS traits more similar to those of the wild types, whereas the mutation m.14484T>C had the strongest tumorigenic properties and the weakest mitochondrial function. The m.11778G>A mutation displayed an intermediate behavior in terms of both tumorigenicity and OXPHOS function (Figures 3 and 4). These results confirm that mtDNA mutations that provoke less severe mitochondrial dysfunction are associated with stronger tumorigenicity and they suggest a correlation within a range of OXPHOS impairment. However, normal mtDNA that allows correct OXPHOS function and mutated mtDNA that provokes severe OXPHOS dysfunction both abolish tumorigenicity, acting as tumor suppressors.

### Mitochondrial mediated tumorigenicity acts through multiples pathways

Although it is accepted that cancer is a multifactorial genetic disease, many studies have focused on the relationship between mitochondria and cancer in cultured tumor cells, pointing to a single parameter as that mainly responsible for tumor formation, development and spreading. Thus, to gain a more complete vision of this process, we analyzed several pathways that may be involved in the differences observed in the tumorigenicity of our cell lines.

### 143B cybrids do not exhibit changes in Ras signaling activity

**Figure 5:**
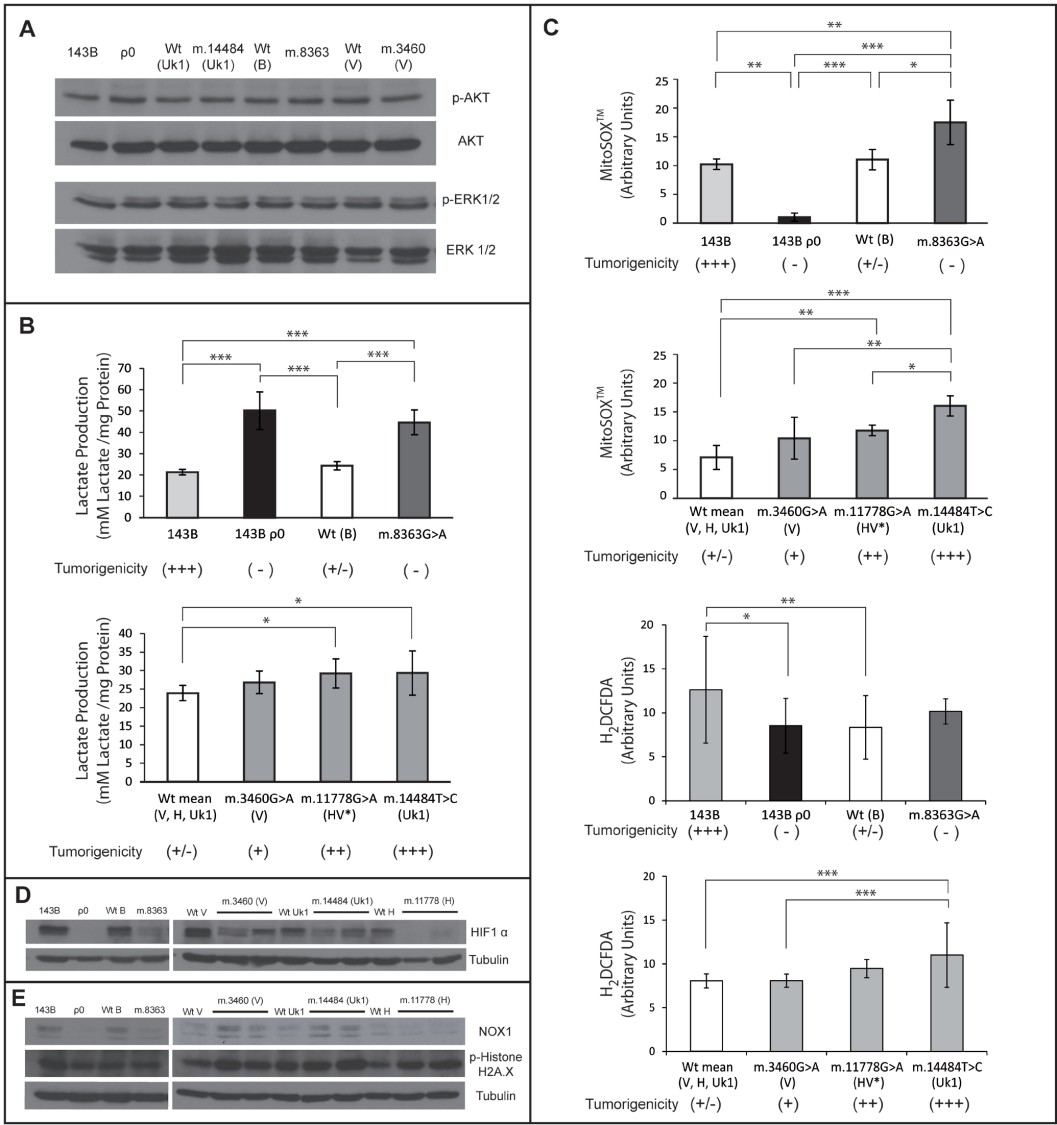
Mitochondrial mediated tumorigenicity acts through multiples pathways (**A**) Western Blots probed for AKT, phospho-AKT, ERK and phospho-ERK 1/2. (**B**) Lactate levels measured in the media after 48h in culture. (C) Mitochondrial superoxide and cytoplasmic ROS levels measured by flow cytometry with the fluorescent probes mitoSOX^TM^ and H_2_DCFDA respectively. In B and (**C)**, wild type clones for the V, H and Uk1 haplogroups were grouped. For the mutants, the bars indicate the mean of two different clones. Measurements for each clone were performed in duplicate in three independent experiments and the error bars represent the standard deviations. The data were analyzed using one way ANOVA and Tukey's multiple comparison test, and a P-value <0.05 was considered significant: *=P≤0.05, **=P≤0.01, ***=P ≤0.001. (**D**) Western Blot of HIF1α with α-tubulin used as a loading control. (**E**) Western Blot of NOX1 and phospho-Histone H2A.X with α-tubulin was used as a loading control.

Oncogenic Ras activates multiple signaling pathways that affect a wide variety of cellular processes that drive tumorigenesis, stimulating proliferation, suppressing apoptosis, and promoting migration and invasiveness, [35]. Since Ras signaling is constitutively activated in the parental 143B cells due to the expression of a K-ras oncogene, we investigated whether mtDNA alterations in our cybrid cell lines affected this pathway. Thus, we analyzed the levels of the phosphorylated extracellular signal regulated kinase (p-ERK1/2) and phosphorylated AKT (p-AKT), both of which are effectors of the two main pathways activated by Ras: the mitogen-activated protein kinase (MAPK) and phosphatidylinositol 3-kinase (PI3K) signaling pathways, respectively. No changes in the expression of p-ERK1/2 and p-AKT were found in the different cybrids with respect to parental 143B cells (Figure 5A), indicating that mtDNA alterations do not modify Ras signaling activity in 143B cells. Since there is no a correlation between the activation of Ras-dependent MAPK or PI3K signaling and the tumorigenicity of the cybrid cell lines, the suppression of tumorigenicity in 143B ρ^0^, m.8363G>A, WT (B, V, H, Uk1) cybrids (Figures 2 and 4) is either independent of Ras, or it occurs downstream of p-AKT and p-ERK1/2 effectors. These results are consistent with the evidence suggesting that activated MAPK signaling in human cancers and mouse models is not always associated with tumorigenesis [36].

### The glycolytic shift correlates with tumorigenicity within a range of OXPHOS function

Increased lactate production is thought to favor tumorigenesis since it is used as a carbon source by neighboring tumor cells, and it induces metabolic reprogramming and angiogenesis. On the other hand, environmental acidosis facilitates invasion by degradation of the extracellular matrix. Indeed, the levels of lactate produced by tumors are correlated with increased metastasis, tumor relapse and poor clinical outcome [37]. Lactate production is higher in our cybrid cell lines harboring mild mtDNA mutations than in the corresponding controls, which may participate in their increased tumorigenic properties (Figure 5B). However, while not tumorigenic, the 143B ρ^0^ and the cell lines containing the severe m.8363G>A mutation produced more lactate, suggesting that additional dominant elements are present in these cases. It is possible that increased glycolytic flux promoted by mtDNA mutations may have a significant impact in reprogramming cancer cells. Indeed, the hypothesis that proto-oncogenes and tumor suppressors genes arose in evolution as metabolic regulators reinforce this idea [3].

### Tumorigenicity induced by mild mtDNA mutations is associated with an increase in mitochondrial and cytoplasmic ROS

There are many cellular sources of reactive oxygen species (ROS) within a cell, they can be generated as an OXPHOS byproduct in the mitochondria as well as in the cytoplasm by other metabolic processes or by the NADPH oxidases in response to specific signals. The role of ROS in tumorigenesis is far from fully understood. It is accepted that tumor formation strongly depends on the amount of ROS [42], affecting (and in turn affected by) different pathways, such as those associated with NOX1 [43], ERK [44], NF-kB [45], AKT [46, 47], HIF1α [38] and SRC [48].

To evaluate the possible correlation between the level of ROS and the tumorigenic capacity of the cybrids we quantified the levels of mitochondrial superoxide anion (O_2_^−^) with MitoSOX^TM^ and the levels of cytoplasmic ROS with H_2_DCFDA.

The m.8363G>A tRNA^Lys^ gene mutation induces high levels of O_2_^−^ compared with 143B ρ^0^ cells (Figure 5C), even though they share similar mitochondrial bioenergetic function (Figure 1). m.8363G>A cybrids barely synthesize 10-15% of mtDNA encoded proteins [18], probably enough to yield ROS generating subcomplexes. On the other hand, 143B ρ^0^ cells, which only synthesize nuclear encoded proteins yielding complex II and a partial complex V (only lacking the two small subunits ATP6-8), are incapable of generating ROS producing subcomplexes. 143B cells containing m.6267G>A generate similar O_2_^−^ levels as cybrids harboring LHON mutations and more O_2_^−^ than control cybrids [15], yet surprisingly equivalent to the O_2_^−^ levels yielded by the specific control WT(B) cybrids (Figure 5C).

Cytoplasmic ROS, showed a similar behavior to mitochondrial superoxide levels. Cells harboring mild or severe mtDNA mutations showed an increase in H_2_DCFDA signal compared with controls and 143B ρ^0^ cells (Figure 5C).

In this cellular system, ROS levels are directly correlated with tumorigenicity unless mitochondrial function is severely diminished, such as when the m.8363G>A mutation is present, which is associated with high ROS production but not tumorigenicity. Thus, while the m.14484T>C cybrid is the strongest ROS producer and exhibited the strongest tumorigenicity, the less tumorigenic m.1178G>A and m.3460G>A cybrids produced less ROS (Figure 5C).

### HIF1α destabilization does not hamper tumorigenicity

Two hallmarks of malignant transformation are a high proliferation rate and increased resistance to hypoxic conditions [2]. Highly proliferative cancer cells in solid tumors usually outgrow their vascular network, limiting oxygen diffusion within the tumor and subjecting it to hypoxic stress. Hypoxia inducible factors (HIFs) promote the metabolic switch towards aerobic glycolysis, neo-vascularization, tumor progression and cell invasion. Thus, HIF1α expression and the downstream activation of a hypoxic stress response are common to many cancers, although surprisingly, HIF1α expression is correlated with lower cancer stage or decreased patient mortality in certain cancers [38]. Thus, the relevance of HIF1α in tumorigenesis remains controversial, with evidence that HIF1α does not markedly affect tumorigenesis [39] or that it may fulfil a central role in this process [25]. Studies with 143B cybrids showed that severe affects on mitochondrial function decrease the stability of HIF1α, which impairs the development of the malignant phenotype [23-25]. This is based on the regulation of HIF1α levels by the activity of prolyl-hydroxylases (PHDs), which are in their turn regulated by the accumulation of TCA cycle intermediates (reflected in the α-ketoglutarate/succinate ratio) [8].

Our tumorigenic cybrid cell lines harboring LHON mutations exhibited lower levels of HIF1α protein than their non-tumorigenic controls (Figure 5D). This may be due to the impaired OXPHOS function being sufficient to promote α-ketoglutarate accumulation and PHD activation. Therefore, in this cell system the levels of HIF1α may contribute to tumorigenicity but not as a driving force. As a matter of fact, HIF1α protein levels were also reduced in non-tumorigenic 143B ρ^0^ and m.8363G>A cybrid cell lines (Figure 5D). These results are to some extent surprising given that the activity of PHDs is inhibited by mitochondrial ROS [38] and therefore, mtDNA mutants that produce higher ROS levels should have more stable HIF1α, which is not the case. This would also indicate that OXPHOS is more important in regulating PHDs than ROS, as suggested when comparing 143B ρ^0^ with m.8363G>A, where the high ROS levels produced by the latter do not rescue the low levels of HIF1α.

Regarding the role of HIF1α in tumorigenesis, our results are consistent with reports that HIF1α knockdown does not affect or even increases the growth of different human carcinomas [39] (references therein). Indeed, HIF1α may even act as a tumor suppressor, since it antagonizes MYC function [40] and it also stabilize p53 [41].

### Tumorigenicity is associated to NOX1 levels

A possible nexus between mitochondrial ROS and tumorigenicity is the induction of NAPDH-oxidase 1 (NOX1) expression as a consequence of retrograde signaling [43]. Members of the NOX family generate superoxide that is rapidly converted into hydrogen peroxide, which in turn may regulate target molecules, acting as a second messenger [49]. Nox1 expression is strongly increased in breast, ovarian and colon tumors [43, 50]. Moreover, its overexpression in cultured cells has been implicated in the stimulation of proliferation [51] and angiogenesis [52], and in the inhibition of apoptosis [50]. The analysis of NOX1 expression (Figure 5E) revealed that NOX1 expression was diminished in control cybrid cell lines and 143B ρ^0^ cells with low ROS levels. On the other hand, LHON mutations that impair but do not abolish OXPHOS (increasing ROS levels) up-regulate NOX1 expression. Strikingly, NOX1 levels are reduced in cell lines with severely diminished mitochondrial function (due to the presence of the m.8363G>A point mutation). Thus, NOX1 levels are correlated with tumorigenicity.

NOX1 expression is related to superoxide levels, except in m.8363G>A cybrids in which the decrease in NOX1 expression triggered by the m.8363G>A mutation is not compensated by an increase in ROS levels (Figure 5). Hence, NOX1 expression appears not only to be regulated by ROS [43]. Therefore, the lack of NOX1 in 143B ρ^0^ cells may be caused by either decreased mitochondrial ROS levels or by a deficiency in mitochondrial function.

Furthermore, the correlation between cytoplasmic and mitochondrial ROS levels could be explained at least in part by the induction of NOX1 expression by the latter: the mitochondrial ROS levels increase NOX1 expression which raises cytoplasmic ROS, suggesting a signal transduction role for NOX1.

In our model, the correlation between NOX1 and tumorigenicity may be related to its importance in maintaining K-RAS mediated transformation. Indeed, increased expression of NOX1 transcripts in colon cancer correlates with activating mutations in K-ras [50]. Moreover, Nox1 RNA interference reverts the K-RAS transformed phenotype, affecting anchorage-independent growth, morphological changes and the production of tumors in athymic mice [53]. Therefore, mild mitochondrial mutants would be more tumorigenic due to the increased NOX1 levels that support the RAS mediated transformation. Confocal microscopy studies revealed that NOX1 localizes to the mitochondria and predominantly, to the perinuclear region [43] where it can induce mutations, genomic instability and changes in the methylation of nuclear DNA in a ROS-dependent manner, favoring neoplastic transformation [54]. In this respect, NOX1 may play a significant role in signal transduction and tumorigenesis enhancing redox signaling to the nucleus.

To evaluate the putative DNA damage induced in the different cell lines, we studied the phosphorylation of histone H2A.X in western blots (Figure 5E). Phosphorylation of the Ser-139 residue in the H2A.X histone variant is an early cellular response to the induction of DNA double-strand breaks (DSBs) and this may reflect cancer-associated genomic instability [55]. H2A.X phosphorylation was stronger in mild mitochondrial mutants (LHON cybrids and parental 143B cells) and it was lower in 143B ρ^0^ cells than in the controls. These results are well correlated with DNA damage and NOX1 expression, with the exception of the m.8363G>A cybrids in which H2A.X is more strongly phosphorylated relative to their low NOX1 expression. It is possible that the higher levels of mitochondrial superoxide in this cell line alone are sufficient to produce DSBs. Moreover, the greater genomic damage in mutants may even be enhanced by deficient repair as a consequence of inhibiting iron-sulphur cluster production [56]. Genomic instability is a driving force for tumorigenesis in which alterations to tumor cell genomes promote the acquisition of further DNA alterations, clonal evolution and finally, tumor heterogeneity, providing a selective advantage to overcome barriers [1]. Therefore, this increased genomic damage could contribute to the tumor phenotype in the mild mutants.

### mtDNA-mediated tumorigenicity correlates with resistance to apoptosis

**Figure 6:**
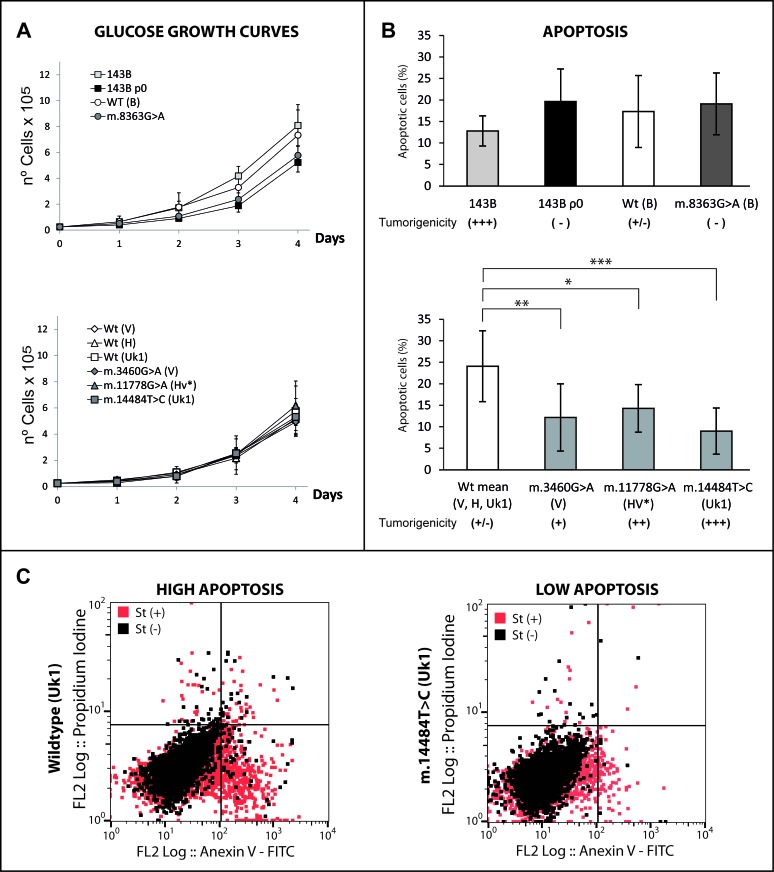
Mild mtDNA mutations increase apoptosis resistance (**A**) Growth curves in 4.5 g/L glucose showing no relevant differences among the cell lines (**B**) Proportion of apoptotic cells after 24 hours in the presence of staurosporine. Wild types clones for the V, H and Uk1 haplogroups were grouped. For mutants, the bars indicate the mean of two different clones. Duplicate measurements for each clone were obtained from three independent experiments and the error bars represent the standard deviations. The data was analyzed using one-way ANOVA and Tukey's multiple comparison test, and a P-value <0.05 was considered significant: *=P ≤ 0.05, **=P ≤ 0.01, ***=P ≤ 0.001. (**C**) Dot plots showing a representative assay for a wild type (high apoptosis) and for a mild mutant (low apoptosis).

Since tumorigenicity reflects the balance between pro- and anti-oncogenic factors, producing a high proliferation and survival phenotype, we first elucidated whether the tumorigenic potential of the cybrids correlated with their proliferative capacity. The growth curves of the cybrids in high-glucose medium were similar (Figure 6A), even though some lines had completely different tumorigenic properties (e.g., the parental 143B and WT(B) cells, Figure 2). Consequently, we hypothesized that the higher tumorigenicity of mild mutant cybrids may be derived, at least partially, from their enhanced survival. Thus, we studied the survival capacity of the cybrids after treatment with staurosporine, a well-known inducer of apoptosis in a wide range of cell lines [57]. Flow cytometry showed tumorigenic cells better resisted staurosporine-induced apoptosis (e.g., m.14484T>C, m.3460G>A and m.11778G>A) than the non-tumorigenic WT (V, H, Uk1: Figure 6B), as did 143B cells with respect to the 143 ρ^0^, WT(B) and m.8363G>A, although in this latter case the differences were not statistically significant. This result at least in part explains the more tumorigenic properties of the mild mutants, indicating that they would be capable of generating more tumors since they are able to better resist the more astringent conditions in the organism. The effect of mtDNA variants on the apoptotic response of the cells has been evaluated in numerous studies with varying results, ranging from mtDNA mutations protecting cells from apoptosis [29, 46] to the contrary [58, 59], possibly due to the different approaches employed.

One possible explanation for the enhanced resistance to apoptosis in mild mutants is related to the dependence of calcium homeostasis on MIMP (Figure 1B, 3B). Mitochondrial calcium intake essentially occurs through the mitochondrial Ca^2+^ uniporter [60]. Since Ca^2+^ is essential for PTP opening, the reduced calcium concentration in the mitochondrial matrix due to the lower MIMP in mild mutant cybrids would impair the triggering of the apoptotic response. Furthermore, the differences in resistance to apoptosis can be explained by the multiple routes modified in response to a retrograde signal that triggers a complex nuclear response. It is likely that the mechanism that confers greater resistance to apoptosis in mild mutants is not activated in 143B ρ^0^ and m.8363G>A cell lines due to other pathways that exert a dominant effect over this process.

## CONCLUSIONS

**Figure 7:**
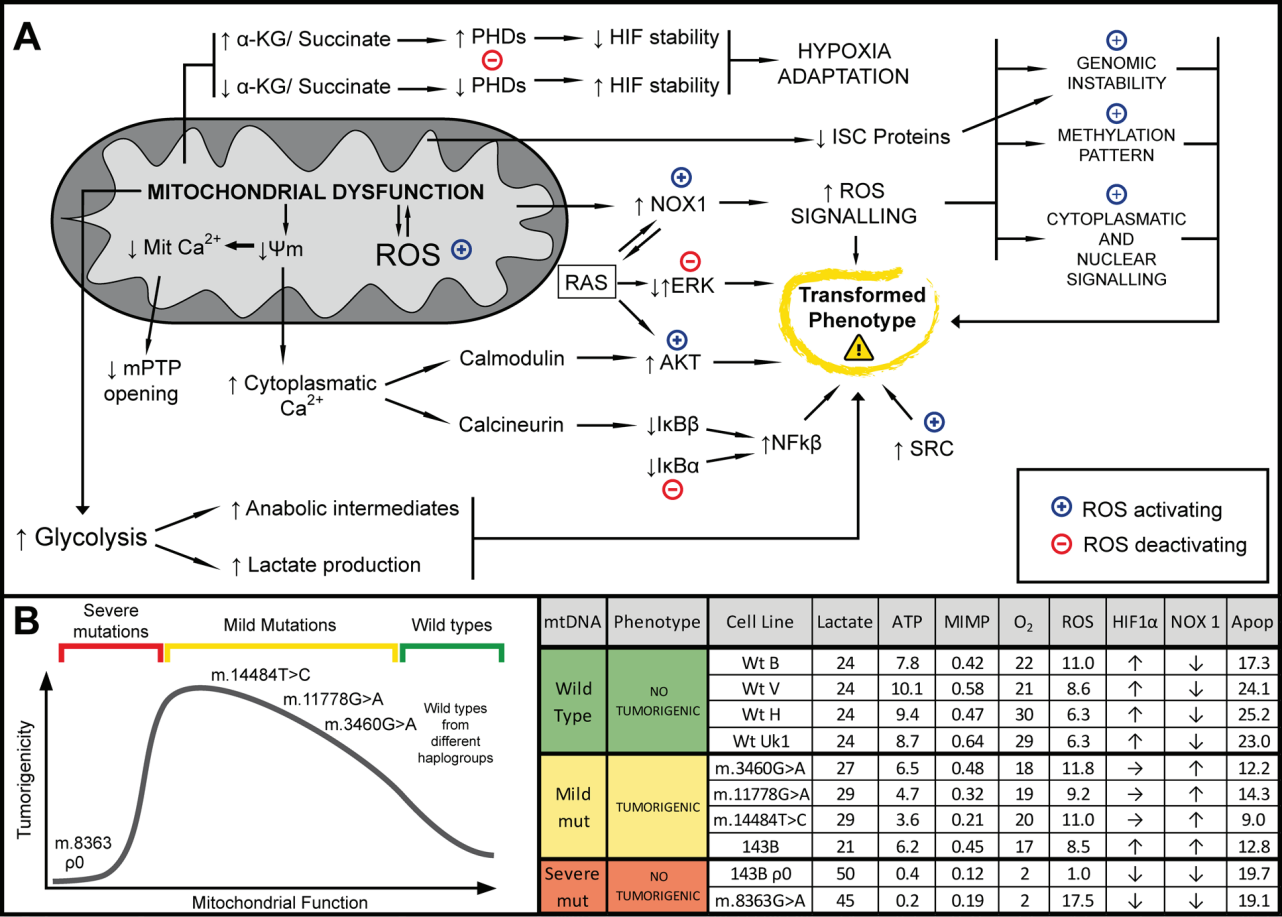
Summary Mitochondrial DNA mild mutations induce tumor formation through complex retrograde signaling. (**A**) Schematic representation of the different pathways involved in the mitochondrial retrograde signal that may affect tumorigenic behavior described here or in the literature. We propose that small changes in OXPHOS function and ROS production trigger a complex mitochondrial retrograde response that ultimately enhances the tumorigenic phenotype: (↑) and (↓) indicate increase/activation or decrease/inhibition, respectively; (+) and (−) indicate increase/activation or decrease/inhibition mediated by ROS, respectively. (**B**) Relationship between mitochondrial function and tumorigenicity found in this study. (**C**) Table summarizing the different mitochondrial parameters measured (Data are the means and the units are shown in the results).

In this study we present convincing evidence that genetic mtDNA variants modulate the tumorigenicity of the 143B osteosarcoma cell line, strongly suggesting that mtDNA rendering a functional or non-functional OXPHOS system does not support tumorigenicity, while mild mtDNA mutations are associated with tumorigenicity. We found two parameters directed related to the tumorigenic potential of the cell lines studied in our experimental model, resistance to apoptosis and increased NOX expression. In addition, the glycolytic rate, ROS levels and Hif1α stability also appear to be correlated with tumorigenicity, yet only within a given range (from unaffected to mildly-affected OXPHOS). Thus, tumorigenic potential probably arises as the result of a balance between several pro- and anti-tumorigenic factors that are modified as a consequence of mitochondrial dysfunction (Figure 7).

Our results suggest that the growth of tumor cells that carry mutated mtDNA that provokes only mild OXPHOS dysfunction will be favored with respect to those cells carrying either normal mtDNA or mutated mtDNA that markedly impairs OXPHOS, both of which act as tumor suppressors. This could explain the numerous and varied mtDNA mutations found in human tumors, most of which give rise to mild mitochondrial dysfunction, as well as the failure to associate a specific mtDNA mutation with a particular tumor type [12]. Any mtDNA mutation leading to mild OXPHOS dysfunction could be selected in tumors.

Our current understanding of how mitochondria influence cancer is still quite basic and much work is needed to better define this relationship. Thus, we must delve deeper into the biology of mitochondria in tumor cells and determine its relationship with the nuclear genome. To decipher the mechanisms involved in the retrograde signals that underlie this relationship will be essential to understand what role the mitochondria play in cancer, where mtDNA could become a diagnostic and prognosis indicator of clinical relevance.

## MATERIALS AND METHODS

### Transmitochondrial cybrid generation and culture conditions

To homogenize nuclear and environmental factors, transmitochondrial cybrids were generated on a osteosarcoma 143B ρ^0^ nuclear background by fusion with platelets, as described elsewhere [19, 20]. Written informed consent to use biological specimens for research purposes was obtained from all patients. The platelet mtDNA was first sequenced on an ABI 3710 sequencer (Applied Biosystems, Foster City, CA) and the variations in the mtDNA were analyzed using the Staden Package [61] to assign the samples to a mitochondrial haplogroup based on the mtDNA variations in the mitomap DB (http://www.mitomap.org/MITOMAP). Individual clones were isolated by limiting dilution, and their mtDNA analyzed by RFLPs and sequencing.

To minimize any potential influence of the nuclear background, all cybrids generated in this work come from a single 143B ρ^0^ clone, and they were isolated and analyzed biochemically, confirming the complete absence of mtDNA by quantitative PCR and in Southern blots (Cruz-Bermúdez et al., manuscript in preparation). In addition, to overcoming the potential variability in clones during cybridization [19, 62], we also analyzed at least two different clones for each cell line in all the experiments.

Cells were grown routinely at 37°C in a humidified atmosphere containing 5% CO_2_, and in 4.5 g/L glucose DMEM supplemented with 10% fetal bovine serum, 50 μg/mL uridine and antibiotics. The cells were maintained in medium containing 2 g/L Glucose and 2.5 g/L Galactose (Glu/Gal DMEM) for 24 hours prior to performing the experiments. Each cell line was checked for mycoplasma contamination during the experiments.

### Measurement of oxygen consumption

The basal respiration of intact cells was measured at 37°C in Glu/Gal DMEM by high resolution respirometry using the Oroboros oxygragh-2k, as described previously [63]. The mean of at least three independent experiments is shown.

### MIMP and ROS measurements

Mitochondrial superoxide and cytoplasmic ROS were assessed using MitoSOX^TM^ red (Invitrogen) and 2′,7′-dichlorodihydrofluorescein diacetate (H_2_DCFDA, Invitrogen) respectively. The mitochondrial inner membrane potential (MIMP) was evaluated as the ratio of tetramethyl rhodamine ester (TMRE, Invitrogen) and MitoTracker Green (MTG, Invitrogen). For these assays, 0.75×10^5^ cells were grown in Glu/Gal DMEM. After addition of the fluorophores (5 μM MitoSOX^TM^, 30μM H_2_DCFDA or 100nM TMRE and 100nM MTG) and incubation at 37°C for 30 min in the dark, the cells were collected in Glu/Gal DMEM and analyzed immediately with a Cytomic FC500 MPL flow cytometer (Beckman Coulter). Forward and side scatter were used to gate the viable population of cells, and the mean fluorescence intensity was determined with MXP software (Beckman Coulter). Experiments were performed in duplicate on at least three independent passages.

### Steady state ATP

Mitochondrial steady state ATP levels were measured in cells incubated previously with 1 mM pyruvate and 5mM 2-deoxy-D-glucose, an irreversible inhibitor of glucokinase, so that cells incubated with it and Pyruvate in the absence of a carbon source synthesizes ATP only in mitochondria. ATP was quantified using the luminometric luciferin-luciferase based method, as described elsewhere [64]. Experiments were performed in duplicate on at least three independent days.

### Lactic acid measurement

Lactate was measured in the medium (Glu/Gal DMEM) in which 10^5^ cells had been plated and cultured for 48 h. Proteins were removed from 50 μL of the medium by adding 100 μl of 8% Percloric Acid and 40% EtOH at 4°C, and centrifuging at 2×10^4^ g for 10 min at 4°C. The supernatants were then frozen until the lactate in 15 μL of each sample, or lactate standard, was measured mixed with 150 μl of assay buffer (0.5M glycine [pH 9.5], 0.2M Hydrazine, 3.4mM EDTA), 100 μl of H_2_0, 5μL of 1100U/mL LDH (Roche) and 30μL of 15 mM NAD^+^. NADH production was evaluated by measuring absorbance at 340nm in a multiplate reader (Synergy HT, Biotek) and it was proportional to the lactic acid concentration in the sample after a 2 h incubation. The lactate concentration was normalized to the total amount of protein measured with the Micro BCA Protein Assay Kit (Thermo Scientific). Assays were performed in triplicate in three independent experiments.

### Cell growth assays

Cell growth was assayed after seeding in 6 well plates at a density of 25,000 cells/well and growing the cells for 4 days in DMEM containing either 4.5 g/L glucose or 0.9 g/L galactose as carbon source. The cells were harvested and counted every 24 hours.

### Measurement of apoptosis

Apoptosis was measured by staining cells simultaneously with Annexin V–FITC (green fluorescence) and the non-vital dye propidium iodide (PI; red fluorescence), discriminating intact (Annexin V-FITC negative, PI negative), early apoptotic (Annexin V-FITC positive, PI negative), late apoptotic (Annexin V-FITC positive, PI positive) and necrotic cells (Annexin V-FITC negative, PI positive). The proportion of necrotic cells was always below 1%.

Flow cytometry analysis was carried out using the MitoStep + FITC Apoptosis Detection Kit, according to the manufacturer's instructions (Immunostep). Briefly, 7.5×10^4^ cells were seeded in Glu/Gal DMEM, apoptosis was induced by exposing them to 500nM Staurosporine for 24 hours and they were analyzed on a Cytomics FC500 MPL flow cytometer (Beckman Coulter). Assays were performed in duplicate in least three independent experiments.

### Western blotting

HIF1, NOX1 and phopho-Histone H2A.X expression was analyzed in cells grown in hypoxic conditions (1% O_2_ for 24 h) using specific antibodies from BD Transduction Laboratories, Santa Cruz Biotechnology (sc-5281) and Cell Signaling (#9718), respectively. To analyze the phosphorylation levels of ERK1/2 and AKT, cells were deprived of serum for 12 h before lysis and the proteins were detected using specific antibodies (Cell Signaling). Tubulin immunodetection (sc-5286, Santa Cruz Biotechnology) was used as a control of protein loading. Appropriate horseradish peroxidase coupled secondary antibodies were used and peroxidase activity was assessed by enhanced chemoluminiscence (Amersham).

### Tumorigenicity assays

All animal experiments were approved by the Animal Care and Use Committees of the UAM and CSIC. Mice were house and handled following institutional guidelines for animal care and in accordance with the standards established in the “National Institutes of Health Guide for the Care and Use of Laboratory Animals”. For tumorigenicity assays, 5×10^6^ cells were injected intradermally/subcutaneously into the two flanks of 8-week-old BALB/c female athymic nude mice (Harlan). The size of the tumors was calculated twice weekly over at least two months based on caliper measurements of two orthogonal diameters. The latency period was defined as the time needed for tumors to reach a size of 0.75 cm^2^.

### Statistics

The results are presented as the mean ± SD, and the statistical analysis was performed using one-way ANOVA and Tukey's multiple comparison test. P-values ≤0.05 were considered significant: *=P≤0.05, **=P≤0.01, ***=P ≤0.001.

## Abbreviations

PTP: Permeability Transition Pore
OXPHOS: Oxidative Phosphorylation
ROS: Reactive Oxygen Species
mtDNA: mitochondrial DNA
LHON: Leber's Hereditary Optic Neuropathy
