# Correction: Enhanced tumorigenicity by mitochondrial DNA mild mutations

**DOI:** 10.18632/oncotarget.27522

**Published:** 2020-03-17

**Authors:** Alberto Cruz-Bermúdez, Carmen G. Vallejo, Ramiro J. Vicente-Blanco, María Esther Gallardo, Miguel Ángel Fernández-Moreno, Miguel Quintanilla, Rafael Garesse

**Affiliations:** ^1^ Instituto de Investigaciones Biomédicas “Alberto Sols”, Consejo Superior de Investigaciones Científicas (CSIC), Universidad Autónoma de Madrid (UAM), Madrid, Spain; ^2^ Departamento de Bioquímica and Centro de Investigación Biomédica en Red en Enfermedades Raras (CIBERER), Facultad de Medicina, UAM, Madrid, Spain; ^3^ Instituto de Investigación Sanitaria Hospital 12 de Octubre (i+12), Madrid, Spain; ^4^ Instituto de Investigación Hospital Universitario La Paz (IdiPAZ), Madrid, Spain

**This article has been corrected:** The Acknowledgements section has been updated as follows:

## ACKNOWLEDGMENTS

This work was supported by the “Instituto de Salud Carlos III” [PI 10/0703 and PI13/00556 to R.G and PI 04/1001 to MAFM]; “Comunidad Autónoma de Madrid” [S2010/BMD-2402 to R.G and S2010/BMD-2359 to MQ); “Fundación Mutua Madrileña” [10.04.02.0064 to MAFM], the Spanish Ministry of Economy and Competitiveness (grant SAF2013-46183-R to MQ) and FEDER funds from the E.U. to R.G. M.E.G. is a staff scientist at the Centro de Investigación Biomédica en Red en Enfermedades Raras (CIBERER), Spain. Flow cytometry analyses were carried out at the “Servicio Interdepartamental de Investigacion” (SIdI) with the excellent technical assistance of Laura Molero Martín.

Original article: Oncotarget. 2015; 6:13628–13643. 13628-13643. https://doi.org/10.18632/oncotarget.3698

